# Diagnostic Problems in Diffuse Axonal Injury

**DOI:** 10.3390/diagnostics10020117

**Published:** 2020-02-21

**Authors:** Sung Ho Jang

**Affiliations:** Department of Physical Medicine and Rehabilitation, College of Medicine, Yeungnam University 317-1, Daemyungdong, Namku, Taegu 705-717, Korea; strokerehab@hanmail.net; Tel.: +82-53-620-4098; Fax: +82-53-625-3508

**Keywords:** diffuse axonal injury, traumatic brain injury, diagnosis, concussion, diffusion tensor imaging

## Abstract

In this study, three problems associated with diagnosing diffuse axonal injury (DAI) in patients with traumatic brain injury are reviewed: the shortage of scientific evidence supporting the 6-hour loss of consciousness (LOC) diagnostic criterion to discriminate concussion and DAI, the low sensitivity of conventional brain MRI in the detection of DAI lesions, and the inappropriateness of the term diffuse in DAI. Pathological study by brain biopsy is required to confirm DAI; however, performing a brain biopsy for the diagnosis of DAI in a living patient is impossible. Therefore, the diagnosis of DAI in a living patient is clinically determined based on the duration of LOC, clinical manifestations, and the results of conventional brain MRI. There is a shortage of scientific evidence supporting the use of the 6-hour LOC criterion to distinguish DAI from concussion, and axonal injuries have been detected in many concussion cases with a less than 6-hour LOC. Moreover, due to the low sensitivity of conventional brain MRI, which can only detect DAI lesions in approximately half of DAI patients, diagnostic MRI criteria for DAI are not well established. In contrast, diffusion tensor imaging (DTI) has been shown to have high sensitivity for the detection of DAI lesions. As DTI is a relatively new method, further studies aimed at the establishment of diagnostic criteria for DAI detection using DTI are needed. On the other hand, because DAI distribution is not diffuse but multifocal, and because axonal injury lesions have been detected in concussion patients, steps to standardize the use of terms related to axonal injury in both concussion and DAI are necessary.

## 1. Introduction 

In 1982, Adams et al. introduced the term diffuse axonal injury (DAI) and later defined it as the presence in the white matter of the brain of microscopic axonal damage produced by mechanical forces [1,2]. Generally, the mechanical forces that induce DAI are associated with sudden acceleration, deceleration, or rotation, of the brain, resulting from unrestricted head movements at the time of the head trauma, and this kind of loading to the brain induces tissue injury characterized by axonal stretching, disruption, and eventual separation of nerve fibers [3,4,5,6]. DAI is characterized histologically by widespread damage to axons, mainly in areas at the border between the cortical gray and white matters such as in the corpus callosum, brainstem, and cerebellum [3,7,8]. DAI is diagnosed in 40%–50% of patients admitted to hospital for traumatic brain injury (TBI), and is closely associated with loss of consciousness (LOC) and a poor outcome after head trauma [8,9].

DAI is a diagnostic term with a pathological meaning; therefore, pathological study by brain biopsy is required to confirm the presence of DAI [1,2,8]. However, performing a brain biopsy to obtain a diagnosis of DAI in living patients is impossible because DAI is not a life-threatening disease, such as is the case for a brain tumor. Therefore, at present, a diagnosis of DAI in a living patient is clinically developed based on LOC duration, clinical manifestations, and results of conventional brain magnetic resonance imaging (MRI) [4,8,10]. Briefly, a diagnosis of DAI is made when a patient has experienced a LOC of more than 6 hours after head trauma, has shown related clinical manifestations, and has DAI lesions on a conventional brain MRI [4,8,10]. However, due to a shortage of scientific evidence supporting the use of the 6-hour LOC criterion to distinguish between concussion and DAI, and the low sensitivity of conventional brain MRI in the detection of DAI lesions, the accuracy of a DAI diagnosis is limited [4,11,12]. In addition, axonal injuries have been detected in many concussion cases with less than 6-hour LOC, and there are opposing opinions regarding the applicability of the term diffuse in DAI [7,11,13,14,15,16,17,18,19,20,21,22].

In this study, I reviewed three diagnostic problems associated with DAI: (1) the shortage of scientific evidence supporting the 6-hour LOC diagnostic criterion to discriminate concussion and DAI, (2) the low sensitivity of conventional brain MRI in the detection of DAI lesions, and (3) the inappropriateness of the term diffuse in DAI.

## 2. Problem 1: The Shortage of Scientific Evidence for the 6-Hour LOC Diagnostic Criterion Separating Concussion and DAI

Head trauma is defined as a concussion when the LOC following the head trauma lasts less than 6 hours, but it is defined as a DAI when the LOC lasts 6 hours or more (Table 1) [11]. However, there is little evidence supporting the efficacy of the 6-hour LOC criterion to distinguish between concussion and DAI considering that axonal injury lesions have been detected in concussion subjects [15,16,18,19,20,21,22]. Gennarelli’s article (1993), which is often cited as the source of the 6-hour criterion, did not cite a reference for the origin of the criterion [11]. Previously, Gennarelli et al. (1982) had investigated the relationship between LOC and DAI in monkeys and reported little different results [18]. In that study, they induced traumatic coma by accelerating the head without direct impact in one of three directions (i.e., whiplash injuries in sagittal, lateral, or oblique directions) and DAI lesions were visible upon pathologic examination in nearly half (7 of 15 monkeys: 46.7%) of the monkeys with an LOC of less than 6 hours after whiplash [18]. Regarding the evidence of DAI lesions in patients with concussion or mild TBI, several studies since the 1960s have, by performing autopsies, detected the presence of DAI lesions in patients with concussion who showed no radiological evidence of brain injury [13,14,23,24]. The renowned Blumbergs et al. study, published by Lancet in 1994, reported the detection of DAI lesions by autopsy in the brain of five patients with concussion, who had died of other causes [14]. Furthermore, DAI lesions were detected in conventional brain MRI scans in 12.5%–30% of patients with mild TBI: Mittl et al. (1994) and Topal et al. (2008) also reported detecting DAI lesions on conventional MRI in 30% and 12.5%, respectively, of patients with mild TBI [15,16]. In addition, accurate estimation of the duration of LOC can be difficult in the clinical field because patients with severe brain injury often require sedation or endotracheal intubation during the acute stage of the TBI.

## 3. Problem 2: The Low Sensitivity of Conventional Brain MRI in the Detection of DAI Lesions

**Figure 1:**
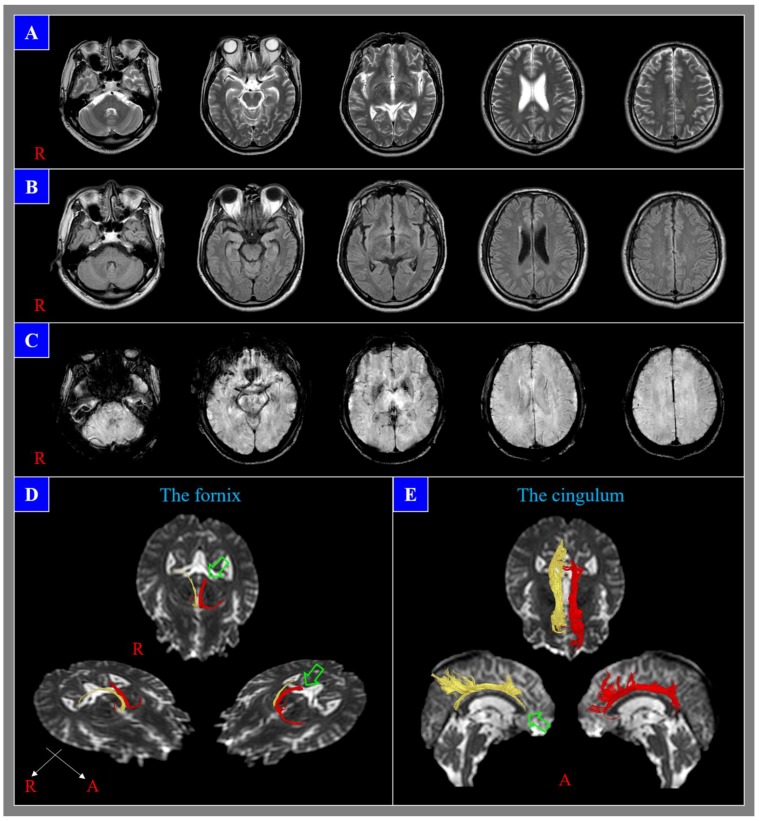
A 54-year-old male who showed loss of consciousness for seven days after a pedestrian–car accident does not show any lesions in (**A**) T2-weighted, (**B**) fluid attenuation inversion recovery (FLAIR), and (**C**) susceptibility-weighted imaging (SWI). However, diffusion tensor tractography results show neural injuries (discontinuations (green arrows): the left fornical crus and right anterior cingulum) of the fornix (**D**) and cingulum (**E**); these injuries are consistent with the subject’s cognitive impairment, which developed after head trauma.

Because of the low-resolution limitation of conventional MRI, it can only detect axonal injury lesions in approximately 50% of DAI cases (Figure 1) [12]. Within the range of DAI lesions, 80% are microscopic or nonhemorrhagic lesions, and approximately 50% of the nonhemorrhagic lesions become normalized with the passage of time [1,3,27,28]. Consequently, it has been suggested that the number of DAI lesions that can be detected by conventional MRI in patients with DAI is only the tip of the iceberg [27,28]. Thus, the inadequate sensitivity of conventional brain MRI is a significant problem in diagnosing DAI, and that inadequacy appears to be mainly attributable to the low-resolution nature of conventional brain MRI. It is estimated that there are at least 10 billion brain cells in the human brain, and a conventional brain MRI scan consists of approximately 300,000 voxels (a voxel represents a point within a three-dimensional plane). Based on these two values, each voxel within a brain MRI scan would specify the status of approximately 30,000 neurons. Based on such low resolution, it is difficult for a conventional brain MRI to accurately reflect the state of damage of a relatively small number of brain cells. Furthermore, it has been shown that brain lesions of several brain diseases other than DAI also cannot be detected by conventional MRI [29,30].

**Figure 2:**
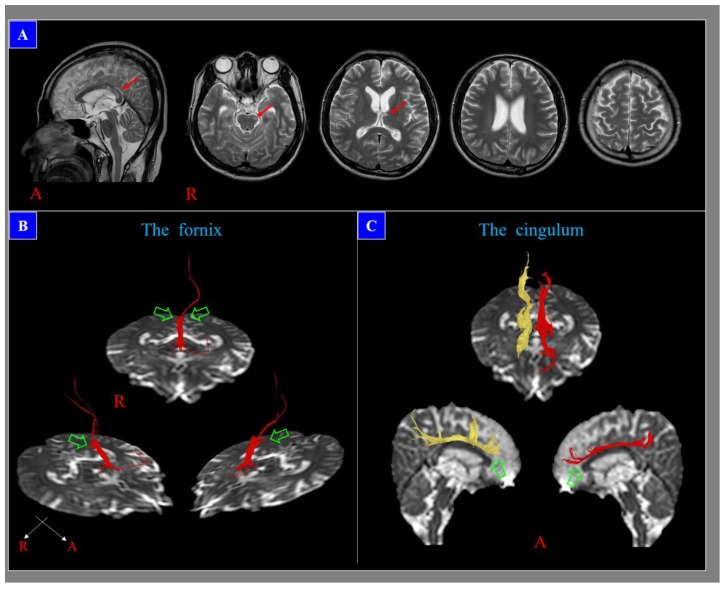
A 28-year-old male who showed loss of consciousness for ten days after a pedestrian–car accident, with diffuse axonal injury lesions (red arrows) in T2-weighted images (**A**). However, diffusion tensor tractography results reveal neural injuries (discontinuations (green arrows): both fornical crura (**B**) and anterior cingulums(**C**)) that are not related to these lesions; these neural injuries are consistent with the subject’s cognitive impairment, which developed after head trauma.

The morphology of DAI lesions is mainly punctate, round, or ovoid, and their size ranges from 1 mm to 15 mm [27,28]. Although a diagnosis of DAI is possible when DAI lesions are detected by conventional brain MRI, often there is insufficient information to determine the extent of the neural injury or develop a prognosis because the DAI lesions are usually small [27,28]. By contrast, diffusion tensor imaging (DTI), a recently introduced imaging technique, has an important advantage as it is able to identify microstructural white matter abnormalities that are not typically detectable on conventional brain MRI [31,32]. DTI provides improved evaluation of the integrity of the white matter of the brain by virtue of its ability to image water diffusion characteristics, which are estimated through various DTI parameters, including fractional anisotropy, mean diffusivity, and tract volume [31,32]. In addition, DTI has been shown to have higher sensitivity for the detection of DAI lesions than that of conventional brain MRI [9,33,34,35,36]. Furthermore, diffusion tensor tractography, which is a derivative of DTI, enables three-dimensional (3-D) visualization and estimation of specific neural tracts [9,36,37,38]. The advantage of diffusion tensor tractography over DTI is that the characteristics of an entire neural tract can be determined by examining the tract’s parameters and analyzing the reconstructed 3-D configuration of the tract [9,36,37,38]. As a result, it can be used to produce detailed 3-D reconstructed imagery of neural tracts, providing marked improvements in detecting the site and extent of DAIs within the examined tracts [9,36,37,38]. DTT abnormality by previous head trauma, concurrent neurological disease, aging, or artifact of DTT should be ruled out [25] (Figure 2).

## 4. Problem 3. The Inappropriateness of the Term Diffuse in DAI

The term “DAI” is somewhat of a misnomer, because the distribution of DAI lesions is not diffuse but multifocal, and such lesions typically involve midline white matter tract areas such as the corpus callosum, brainstem, and cerebellar peduncles [1,2,17]. Therefore, effort has been made to use terms such as traumatic axonal injury or diffuse traumatic axonal injury to correct for the inappropriateness of the term “diffuse” in DAI and to emphasize the etiology of the axonal injury in head trauma [1,2,7,17]. Often, patients diagnosed with the traditional definition of DAI are in a profound coma from the onset of injury, resulting in a poor outcome [1,39]. With the development of improved neuroimaging techniques, such as DTI, that provide more detail than conventional MRI, patients have been shown to have more restricted distribution patterns of axonal injuries than those seen in classic DAI. The term traumatic axonal injury has been used for patients exhibiting these more limited axonal injury patterns, and, in practice, traumatic axonal injury rather than DAI has been used in mild injury cases [17,19,20,21,22,39].

## 5. Conclusions

In this review, I describe three diagnostic problems associated with DAI: the shortage of scientific evidence supporting the 6-hour LOC diagnostic criterion to discriminate concussion and DAI; the low sensitivity of conventional brain MRI in the detection of DAI lesions; and the inappropriateness of the term diffuse in DAI. Pathological study by brain biopsy is required to confirm DAI, but performing a brain biopsy for diagnosing DAI in living patients is impossible; therefore, clinical diagnosis of a DAI is currently based on the duration of LOC, relevant clinical manifestations, and conventional brain MRI results [1,2,4,8,10]. The use of the 6-hour LOC criterion to distinguish between concussion and DAI is not well supported, as axonal injuries have been detected in many concussion cases with less than 6-hour LOC [13,14,15,16,19,20,21,22]. Diagnostic criteria for DAI have not been established, due to the low sensitivity of conventional brain MRI for the detection of DAI lesions and the absence of studies into MRI sensitivity and specificity related to MRI criteria for diagnosing DAI [1,2,4,8,10]. The recently introduced DTI method has been shown to have higher sensitivity for the detection of DAI lesions than conventional brain MRI; however, further studies on the establishment of DTI-based diagnostic criteria for DAI are necessary [9,33,34,35,36]. Many studies have reported that axonal injury is a consistent feature of all TBI and have shown that the distribution and number of damaged axons increase with trauma severity from mild to moderate and finally severe TBI [40,41]. Therefore, DTI abnormality by previous head trauma should be ruled out [25]. Further studies to overcome the limitations of DTI that are related to artifact and reliability are warranted. Lastly, because the distribution of DAI is not diffuse but multifocal, and axonal injury lesions have been detected in concussion patients, steps to standardize the use of terms related to axonal injury in both concussion and DAI are necessary [13,14,15,16,19,20,21,22,39]. 

## Figures and Tables

**Table 1:** Classification of traumatic brain injury [25,26].

**Patho-Anatomy**			
Diffuse		Focal	
Concussion		Contusion	
Traumatic axonal injury/diffuse axonal injury		Penetrating	
Explosion		Hematoma	
Abusive head trauma		- Epidural	
		- Subarachnoid	
		- Subdural	
		- Intraventricular	
		- Intracerebral	
**Severity of Head Trauma**			
	LOC	PTA	GCS
Mild:	≤30 min	≤24 h	13–15
Moderate:	>30 min, ≤24 h	>24 h, ≤7 days	9–12
Severe:	>24 h	>7 days	3–8

LOC: loss of consciousness, PTA: post-traumatic amnesia, GCS: Glasgow coma scale. (reprinted with permission from Jang, S.H., Traumatic Brain Injury. *In.Tech.* **2018;** 137–154).

## References

[1] Adams J.H., Graham D.I., Murray L.S., Scott G. (1982). Diffuse axonal injury due to nonmissile head injury in humans: An analysis of 45 cases. Ann. Neurol..

[2] Adams J.H., Doyle D., Ford I., Gennarelli T.A., Graham, D.I., McLellan D.R. (1989). Diffuse axonal injury in head injury: Definition, diagnosis and grading. Histopathology.

[3] Chung S.W., Park Y.S., Nam T.K., Kwon J.T., Min B.K., Hwang S.N. (2012). Locations and clinical significance of nonhemorrhagic brain lesions in diffuse axonal injuries. J. Korean Neurosurg Soc..

[4] Smith D.H., Meaney D.F., Shull W.H. (2003). Diffuse axonal injury in head trauma. J. Head Trauma. Rehabil..

[5] Povlishock J.T., Christman C.W. (1995). The pathobiology of traumatically induced axonal injury in animals and humans: A review of current thoughts. J. Neurotrauma.

[6] Buki A., Povlishock J.T. (2006). All roads lead to disconnection?--traumatic axonal injury revisited. Acta. Neurochir (Wien).

[7] Maxwell W.L., Povlishock J.T., Graham D.L. (1997). A mechanistic analysis of nondisruptive axonal injury: A review. J. Neurotrauma.

[8] Meythaler J.M., Peduzzi J.D., Eleftheriou E., Novack T.A. (2001). Current concepts: Diffuse axonal injury-associated traumatic brain injury. Arch. Phys. Med. Rehabil..

[9] Grassi D.C., Conceicao D.M.D., Leite C.D.C., Andrade C.S. (2018). Current contribution of diffusion tensor imaging in the evaluation of diffuse axonal injury. Arq. Neuropsiquiatr.

[10] Jing S., Ju Y., He Y., He M., Mao B. (2001). Clinical features of diffuse axonal injury. Chin. J. Traumatol..

[11] Gennarelli T.A. (1987). Cerebral concussion and diffuse brain injuries.

[12] Humble S.S., Wilson L.D., Wang L., Long D.A., Smith M.A., Siktberg J.C., Mirhoseini M.F., Bhatia A., Pruthi S., Day M.A. (2018). Prognosis of diffuse axonal injury with traumatic brain injury. J. Trauma Acute Care Surg..

[13] Oppenheimer D.R. (1968). Microscopic lesions in the brain following head injury. J. Neurol. Neurosurg Psychiatry.

[14] Blumbergs P.C., Scott G., Manavis J., Wainwright H., Simpson D.A., McLean A.J. (1994). Staining of amyloid precursor protein to study axonal damage in mild head injury. Lancet.

[15] Mittl R.L., Grossman R.I., Hiehle J.F., Hurst R.W., Kauder D.R., Gennarelli T.A., Alburger G.W. (1994). Prevalence of MR evidence of diffuse axonal injury in patients with mild head injury and normal head CT findings. AJNR Am. J. Neuroradiol..

[16] Topal N.B., Hakyemez B., Erdogan C., Bulut M., Koksal O., Akkose S., Dogan S., Parlak M., Ozguc H., Korfali E. (2008). MR imaging in the detection of diffuse axonal injury with mild traumatic brain injury. Neurol. Res..

[17] Johnson V.E., Stewart W., Smith D.H. (2013). Axonal pathology in traumatic brain injury. Exp. Neurol..

[18] Gennarelli T.A., Thibault L.E., Adams J.H., Graham D.I., Thompson C.J., Marcincin R.P. (1982). Diffuse axonal injury and traumatic coma in the primate. Ann. Neurol..

[19] Shenton M.E., Hamoda H.M., Schneiderman J.S., Bouix S., Pasternak O., Rathi Y., Vu M.A., Purohit M.P., Helmer K., Koerte I. (2012). A review of magnetic resonance imaging and diffusion tensor imaging findings in mild traumatic brain injury. Brain Imaging Behav..

[20] D’Souza M.M., Trivedi R., Singh K., Grover H., Choudhury A., Kaur P., Kumar P., Tripathi R.P. (2015). Traumatic brain injury and the post-concussion syndrome: A diffusion tensor tractography study. Indian J. Radiol Imaging.

[21] Lee S.J., Bae C.H., Seo J.P., Jang S.H. (2019). Diagnosis of Tinnitus Due to Auditory Radiation Injury Following Whiplash Injury: A Case Study. Diagnostics (Basel).

[22] Jang S.H., Lee H.D. (2019). Diagnostic Approach to Traumatic Axonal Injury of the Spinothalamic Tract in Individual Patients with Mild Traumatic Brain Injury. Diagnostics (Basel).

[23] Jang S.H. (2016). Diagnostic history of traumatic axonal injury in patients with cerebral concussion and mild traumatic brain injury. Brain NeuroRehabil..

[24] Bigler E.D. (2004). Neuropsychological results and neuropathological findings at autopsy in a case of mild traumatic brain injury. J. Int. Neuropsychol. Soc..

[25] Jang S.H., Gorbunoy N. (2018). Traumatic axonal injury in mild traumatic brain injury. Traumatic Brain Injury.

[26] Hill C.S., Coleman M.P., Menon D.K. (2016). Traumatic axonal injury: Mechanisms and translational opportunities. Trends Neurosci..

[27] Gentry L.R., Godersky J.C., Thompson B. (1988). MR imaging of head trauma: Review of the distribution and radiopathologic features of traumatic lesions. AJR Am. J. Roentgenol..

[28] Katzman G.L., Osborn A.G., Salzman K.L., Barkovich A.J. (2010). Trauma; Diffuse axonal injury. Diagnostic Imaging: Brain.

[29] Jang S.H., Kim S.H., Lim H.W., Yeo S.S. (2014). Injury of the lower ascending reticular activating system in patients with hypoxic-ischemic brain injury: Diffusion tensor imaging study. Neuroradiology.

[30] Yu F.F., Chiang F.L., Stephens N., Huang S.Y., Bilgic B., Tantiwongkosi B., Romero R. (2019). Characterization of normal-appearing white matter in multiple sclerosis using quantitative susceptibility mapping in conjunction with diffusion tensor imaging. Neuroradiology.

[31] Basser P.J., Mattiello J., LeBihan D. (1994). Estimation of the effective self-diffusion tensor from the NMR spin echo. J. Magn. Reson..

[32] Mori S., Crain B.J., Chacko V.P., van Zijl P.C. (1999). Three-dimensional tracking of axonal projections in the brain by magnetic resonance imaging. Ann. Neurol..

[33] Huisman T.A., Schwamm L.H., Schaefer P.W., Koroshetz W.J., Shetty-Alva N., Ozsunar Y., Wu O., Sorensen A.G. (2004). Diffusion tensor imaging as potential biomarker of white matter injury in diffuse axonal injury. AJNR Am. J. Neuroradiol..

[34] Brandstack N., Kurki T., Tenovuo O. (2013). Quantitative diffusion-tensor tractography of long association tracts in patients with traumatic brain injury without associated findings at routine MR imaging. Radiology.

[35] Lee J.W., Choi C.G., Chun M.H. (2006). Usefulness of diffusion tensor imaging for evaluation of motor function in patients with traumatic brain injury: Three case studies. J. Head Trauma Rehabil..

[36] Wang J.Y., Bakhadirov K., Devous M.D., Abdi H., McColl R., Moore C., Marquez de la Plata C.D., Ding K., Whittemore A. (2008). Diffusion tensor tractography of traumatic diffuse axonal injury. Arch. Neurol..

[37] Jang S.H., Seo Y.S. (2020). Difference between injuries of the corticospinal tract and corticoreticulospinal tract in patients with diffuse axonal injury: A diffusion tensor tractography study. Int. J. Neurosci..

[38] Choi G.S., Kim O.L., Kim S.H., Ahn S.H., Cho Y.W., Son S.M., Jang S.H. (2012). Classification of cause of motor weakness in traumatic brain injury using diffusion tensor imaging. Arch. Neurol..

[39] Saatman K.E., Duhaime A.C., Bullock R., Maas A.I., Valadka A., Manley G.T., Workshop Scientific T., Advisory Panel M. (2008). Classification of traumatic brain injury for targeted therapies. J. Neurotrauma.

[40] Povlishock J.T., Erb D.E., Astruc J. (1992). Axonal response to traumatic brain injury: Reactive axonal change, deafferentation, and neuroplasticity. J. Neurotrauma.

[41] Wallace E.J., Mathias J.L., Ward L. (2018). Diffusion tensor imaging changes following mild, moderate and severe adult traumatic brain injury: A meta-analysis. Brain Imaging Behav..

